# Comparison of ethical judgments exhibited by clients and ethics consultants in Japan

**DOI:** 10.1186/1472-6939-15-19

**Published:** 2014-03-04

**Authors:** Noriko Nagao, Yasuhiro Kadooka, Atsushi Asai

**Affiliations:** 1 Department of Nursing, Kobe University Graduate School of Health Sciences 7-10-2 Tomogaoka, Suma-ku, Kobe, Hyogo 654-0142, Japan; 2 Department of Bioethics, Kumamoto University Graduate School of Medical Science, Kumamoto, Japan

**Keywords:** Clinical ethics consultation, Ethical judgment, Ethical reasoning, Clinical ethics, Quality of healthcare

## Abstract

**Background:**

Healthcare professionals must make decisions for patients based on ethical considerations. However, they rely on clinical ethics consultations (CEC) to review ethical justifications of their decisions. CEC consultants support the cases reviewed and guide medical care. When both healthcare professionals and CEC consultants face ethical problems in medical care, how is their judgment derived? How do medical judgments differ from the ethical considerations of CECs? This study examines CECs in Japan to identify differences in the ethical judgment of clients and CEC consultants.

**Methods:**

The CEC request and response documents of all 60 cases reviewed across Japan between October 2006 and the end of October 2011 were classified in terms of the presence of decisional capacity in the patient. We conducted a qualitative content analysis of the differences in reasoning between client and CEC consultants. Reasoned judgments were verified in individual cases to classify the similarities or differences of opinion between CEC clients and teams.

**Results:**

As the result of classification of the decisional capacity and the difference of opinion regarding medical care, the most frequent category was 25 cases (41.7%) of “uncertain decisional capacity,” and 23 cases (38.3%) of “withholding of decision-making.” A chi-square analysis was performed on presence of decisional capacity and agreement in decision-making, yielding a statistically significant difference (*p* < 0.05). The CEC consultants’ reasoning was based on “patient’s preference was ambiguous,” “validity of family as a surrogate,” “estimation of patient preference,” and “patient’s best interest,” whereas the CEC client’s reasoning was based on “consistent family preference was shown/not shown” and “appropriate therapeutic methods to manage patient safety.”

**Conclusion:**

Differences in opinions were found in cases classified according to decisional capacity. Furthermore, the reasoning behind judgments differed between CEC clients and CEC consultants. The reasoning of CEC consultants was critical and reflective, while for clients it was situational and pragmatic.

## Background

Clinical ethics consultation (CEC) is a conflict resolution mechanism for parties involved in value-laden healthcare disputes and it is well accepted in the West [1-4]. As one activity of Hospital Ethics Committees, CEC is conducted in team or individual formats. Hospital systems in Asia value hierarchical working relationships, creating Asia-specific issues for the spread and implementation of CEC [5]. However, ethics committees are becoming more common because having a resolution mechanism for clinical ethical disputes is now an accreditation requirement for hospitals in Japan [6]. They have both research and clinical ethics roles, yet with only 24.7% of teaching hospitals in Japan providing CEC, clinicians still have little support in clinical ethics [7].

Healthcare professionals rely on CEC for issues, such as decisions about end-of-life care, decisional capacity, and choice of legal surrogates, not only in Western nations, but also in Japan [4,7,8]. It can negate the need for litigation, mediate conflict between healthcare professionals and patients or their surrogates, achieve objectivity through a third party, and identify morally acceptable alternative therapies [3,4,6,8,9]. However, healthcare professionals must constantly make medical judgments based on ethical considerations [10-12]. They also have their own perception of problems and weighted values regarding medical care. They also face conflict among all parties involved and struggle with decision making. Because their judgments are affected by medical and practical pitfalls, these are balanced by ethical reflective perspectives. Their judgments differ from the judgments that ethics consultation supports.

Ethics consultants typically prioritize patient preferences. Other ethical considerations include resource allocation, legal constraints, and family preferences in clinical settings [10-13]. Consultants are expected to have expertise in patient care ethics and law; however, clinical judgments of the condition of a patient with chronic disease often depend on traditional social norms. A previous study by the authors suggests that judgments about end-of-life care in the case of Alzheimer’s disease differ between US and Japanese ethics consultations. Japanese consultants tend to prioritize the patient’s best interests, while US consultants tend to prioritize patient preferences with best interests being a secondary consideration [14]. In Japan, the focus is on evaluating the uncertain clinical state of chronic diseases and the limitations of the end-of-life healthcare system, and on making various value judgments related to futility [14]. In brief, even if the framework for ethical discussion is identical, there may be variations in values, regions, systems, and laws that affect professionals’ priorities and procedures within that framework, as well as differences in how they evaluate the relevance of case-specific information.

When healthcare professionals face ethical problems in medical care, how is their judgment derived? How do CEC consultants understand the cases reviewed and guide medical care, and does this process differ from that of healthcare professionals? Few studies have compared the ethical judgments of healthcare professionals and CEC consultants within the same country. This study aimed to examine differences in judgment and reasoning between healthcare professionals and ethics consultants in handling CEC client requests.

### Summary of CEC activities in this study

The main role of ethics committees in Japanese hospitals is to review clinical research; however, committees have recently been developed to review clinical ethics. Until recently, the priority of reviewing research ethics has been higher than that of clinical ethics, particularly in larger hospitals. However, even in small- and medium-sized hospitals, the organization is unable to provide both. The Clinical Ethics Support/Education Project has provided CEC in support of hospitals in Japan since 2006 for free, by review and consideration of requests from healthcare professionals as well as patients and their families. Clinical ethics consultants under the Project identify and analyze ethical issues in clinical practice and then provide appropriate ethical advice to clients. They explore a potential ethical issue in requested cases, and provide appropriate study results that affect value judgments in clinical settings. Initially, these activities were part of an experiment approved by the Ethical Review Board at Kumamoto University, but now, they have become a voluntary and practical form of support. Requests are sent to the Project by e-mail or fax from healthcare providers, patients, or patient surrogates (CEC clients). CEC consultants, drawn from a pool of 25 volunteers from the fields of clinical ethics, medicine, nursing, law/ethics, and psychology, provide independent support and advice. One CEC team might typically consist of three to five members from various specialties, workplaces, genders, or age groups, depending on preferences emailed or faxed from healthcare providers and, less frequently, patients and their surrogates [15]. This support is open to the public through the media and a website, and consultants respond to requests from all over Japan. Cases are sent to the Administration of the Department of Bioethics of the Graduate School of Medical Science at Kumamoto University. Anyone can request a case review.

Corresponding through e-mail, the consultation team discusses a given request and then collaboratively creates a document detailing their advice for the CEC client. This is usually done within one week, but it can be done within one to five days in special cases. A team leader is familiar with the ethical problems related to a particular case, and the consultant members of the team discuss the issues in writing. Typically, one week is spent building consensus. The final version of the advice, based on team member agreement, is then returned to the client by the administration.

## Method

The document included the results of the consultants’ discussion and the ethical grounds upon which the reasoning and conclusions were based. Between October 2006 and October 2011, a total of 60 requests were handled across Japan: all 60 cases were both ongoing cases and cases in past. Qualitative content analysis was retrospectively performed on the request and response documents of all 60 cases [16]. CEC client information was made anonymous by the administration, and then the CEC team judged the cases according to the information solely present in the request form. Thus, we judged not only the work but also analyzed information from either the request forms sent to the CEC team or the recommendation forms sent to the CEC clients as secondary use. This study was approved by the Research Ethics Committee of the Kobe University, Graduate School of Health Sciences.

Of the 60 cases, CEC clients were most frequently a medical doctor and medical team (16 cases), followed by nurses (7 cases), staff in other clinical departments (4 cases), ethics committees in hospitals (3 cases), patient families (4 cases), a patient (1 case), a patient’s friend (1 case), a guardian (1 case), and an unidentified individual within the clinic (5 cases). In order to refer cases to the project for consultation, CEC clients send a request form, which documents the ethical issues and the context of the case, outlining the medical aspects, patient and family preferences, patient quality of life, and social utility. The CEC consultation team’s aim is to broadly recommend ways to address the issues.

### Analysis

In the study, the content of all requests were classified by theme and the request and response documents were evaluated for presence of patient decisional capacity. To understand the contents of the request and response documents, case analysis with discussion was performed by the authors [16]. The judgment in this study was defined as the judgment that the CEC client considered for the case, the judgment that the CEC team considered for the case, and whether the judgment of the CEC client was supported. Presence of a patient’s decisional capacity is an important aspect of decision making in clinical settings. When a patient does not have the decisional capacity to allow for their own autonomy, it is difficult for a third party to estimate a patient’s best interests. Thus, conflict can arise from the differences in values among stakeholders, and most often leads to all parties being dissatisfied. Cases in which patients showed definite preferences with decisional capacity were classified as “with decisional capacity.” Cases in which patients indicated a clear lack of decisional capacity were classified as “without decisional capacity.” Cases in which there was insufficient information to demonstrate decisional capacity were classified as having “uncertain decisional capacity.” Finally, cases in which decisional capacity was not required were classified as “no relationship.”

The reasoning leading to both the CEC client’s and the CEC team’s judgments was extracted from each document and coded according to the information provided in the request form. Initially, two of the authors (NN, YK) independently coded the data by keyword, and then reviewed the sets of keywords and categories along with the initial data separately. The coding pertain to clinical condition, patients’ capacity, family perspectives, and legal status. The principal author (NN) examined the classification and the coding agreement as follows: existence or non-existence of patients’ capacity and/or preferences, agreement or disagreement of the CEC clients’ and teams’ opinions. Then, two coders (NN, YK) reviewed the research question and literature review. The coders independently compared to ensure coding agreement. Another coauthor (AA) supervised the analysis. All authors discussed any discrepancy regarding the interpretation of the data until they reached a consensus [17].

We analyzed the written opinions and the points of dispute regarding medical care decisions as discussed by the CEC team. In doing so, we classified these decisions according to the similarities or differences of opinion between CEC clients and teams. For decisions regarding medical care, the categories were “agreed,” “disagreed,” “partially agreed,” (opinions were in agreement but reasoning was not), “withheld” (ambiguous CEC client opinion or no CEC team decision, including substantial advice or specific recommendations on medical care or an ethically preferable policy), and “other.” Co-authors reviewed the coding and categorization independently. We also classified CEC team opinions regarding patient preferences for care; these categories were “for,” “against,” “undetermined” (decisional capacity present but patient preference ambiguous), and “not applicable” (decisional capacity absent).

The classified data were entered into the statistical software SPSS Version 17. Chi-square tests were then used to compare the presence of patient decisional capacity, and differences of opinion between judgments of CEC clients and CEC teams. Significance was set at *p* < 0.05.

## Results

The contents of the requests for all 60 cases were classified by theme; the most common theme was life-and-death decisions for 27 cases without decisional capacity. There were also nine cases of treatment refusal among patients (including the handling of advance directives by patients who had a decisional capacity at the time), three cases regarding how to explain a Do Not Attempt Resuscitation (DNAR) order, and three cases regarding the appropriateness of truth telling in diagnosis and prognosis. Many other cases with various themes were also requested (Table 1).

**Table 1:** Cases of requested ethics consultation (N = 60)

**Contents of request**	**Number of cases**
Judgment about life and death of patients who lacked decision-making capacity	27
Treatment refusal by patients (including cases in which the patients had decision-making capacity at that time)	9
Method of explanation and consent regarding the DNAR	3
Appropriateness of a truth-telling about diagnosis or prognosis	3
Terminal sedation	2
Patient’s demand for useless treatment	2
Privacy/disclosure of private information	2
Appropriateness of neonatal care	2
Problematic behavior of patients and families	2
Judgment about home terminal care	2
Appropriateness of using a placebo as a clinical treatment	1
How to hold ethical committees/conferences	1
Abortion	1
Occupational safety of health-care provider	1
Patient refusal of necessary consultation	1
Refusal of surgical treatment for patient with psychiatric disorder	1

(As of October 31, 2011).

No additional data.

Decisional capacity was categorized from the request and response documents of all 60 cases. The most frequent case categorization was “uncertain” (25, 41%), followed by “with decisional capacity” (18, 30%), “without decisional capacity” (12, 20%), and “irrelevant” (5, 8.3%).

The opinions regarding medical care between the CEC team and CEC client were also classified. The most frequent cases were “withheld” (23, 38.3%), followed by “partially agreed” (16, 26.7%), “agreed” (10, 16.7%), “disagreed” (9, 15%), and “others” (2, 3.3%; Table 2).

**Table 2:** Classification list of patients’ decisional capacity, agreement of opinion about medical care between the clinical ethics consultation (CEC) client and team, and approval of the patient preference from the consultation team

	**Number of patients**	**%**
Patient’s decisional capacity	(n = 60)	
With decisional capacity	18	30
Without decisional capacity	25	41.7
Uncertain	12	20
No relationship	5	8.3
Agreement of opinion about medical care between the CEC client and CEC team	(n = 60)	
Agreed	10	16.7
Disagreed	9	15
Partially agreed	16	26.7
Withheld	23	38.7
Other	2	3.3
Approval of the patient preference from the CEC team	(n = 55)	
Against patient preference	4	7.3
For patient preference	9	16.4
Undetermined because of ambiguous patient preference	19	34.5
Not applicable because the patient had no decisional capacity	23	41.8

Variations in opinion related to the presence of decisional capacity were examined. The cases were assigned to two groups, one for decisional capacity (“with decisional capacity” vs. “without decisional capacity/uncertain decisional capacity”) and the other for variations in opinion between CEC clients and teams (“agreed/partially agreed” vs. “disagreed/withheld”). These groups were significantly different statistically, as revealed by a chi-squared analysis (*p* < 0.05; Table 3).

**Table 3:** Judgment agreement by presence of patient’s decisional capacity

		**Agreement/disagreement of the opinion**			
		**Disagreed/withheld**	**Agreed/partially agreed**	**Total**	
Decisional capacity	Without/uncertain	23	14	37	*p* = 0.02
	With	5	12	17	
Total		28	26	54	

A chi-squared was used to compare percentages between the patient’s decisional capacity and agreement/disagreement of the opinion. *p* < 0.05 is the statistically significant difference.

The categorizations for the relevant reasons behind CEC-client and CEC-team judgments were assigned to the agreed and disagreed groups (Table 4). We described the content of the judgments, then a typical case, and the reasons behind the judgments.

**Table 4:** Approval of patient preference by the clinical ethics consultation team for the “with decisional capacity” group (n = 18)

	**Number of cases**	**%**
Approval of patient preference by the CEC team		
Against patient preference	3	16.7
In approval of patient preference	7	38.9
Undetermined because of ambiguous patient preference	8	44.4

For seven of the nine cases of “without decisional capacity” in the disagreed group, CEC client judgments were based on the following:

1. Poor prognosis

2. Absence of advance directive

3. Ambiguous patient preference

4. Presence/absence of consistent family preference

5. Family expectations for patient recovery

6. Most appropriate therapeutic methods to manage patient safety regardless of patient preference, instead of respect for patient preference

Additionally, one particular request (involving privacy and confidentiality) was based on the following:

7. Interpretation differences regarding privacy protection between healthcare providers

In contrast, CEC team judgments were based on the following:

1. Poor prognosis

2. Ambiguous patient preference

3. Validity of family as a surrogate

4. Patient preference based on estimation of patient’s interest and harm

5. Severe patient suffering

6. Variation and evaluation of QOL associated with treatment

7. The patient’s best interests

Additionally, one particular request (involving privacy and confidentiality) was based on the following:

8. Physical restraint criteria

9. Right to control information

### An example of a typical case (A) of “without decisional capacity” in the disagreed group

A patient in their 80s with multiple intractable skin ulcers at the periphery of the limbs had received antibiotic and steroid-pulse therapy for rapidly progressive interstitial pneumonia. The patient was put on a respirator due to a deteriorating condition and was then extubated upon improvement. However, when recurrent dyspnea occurred, the patient was re-intubated. Three weeks later, the family expressed their aversion to prolonged treatment using mechanical ventilation via a tracheostomy without the possibility of improvement in the patient’s respiration. When extubated previously, the patient had requested to be allowed to die. If dyspnea occurred again, the patient would need to be re-intubated via tracheostomy in order to have a chance to survive. On the other hand, the family does not want re-intubation. The patient’s current preference is unclear. How should this patient be treated if the absence of a tracheostomy leads to ethical problems?

[CEC client opinion]

A tracheostomy and intubation are preferable when the patient’s respiration worsens again.

[Advice from the CEC team]

1. Even with temporary improvement, it will be difficult to save the patient’s life. It is important to confirm and respect family preferences and, if it can be confirmed, the patient’s preference as well.

2. The advantages and disadvantages of frequent ventilator use should be identified and evaluated.

3. Without intubation, sedation is required to relieve pain, although this is subject to family consent. It is very important to discuss life-prolonging therapies in view of the patient’s pain, QOL, and comfort.

To summarize the results of Case A, the reasons for the CEC client’s opinion included the seriousness of dyspnea complicated by interstitial pneumonia, ambiguousness of patient preference, family aversion to re-intubation, and appropriate risk management. Therefore, the client’s opinion was that a tracheostomy and intubation were preferable when the patient’s respiration worsens. In contrast, reasons for CEC team’s opinion included terminal prognosis, invasive procedures, unknown patient preference, and evaluation of the risks and benefits of re-intubation. In sum, the CEC team’s advice was comfort care.

Both the CEC client and CEC team deliberated from the common perspective of the patient’s physical condition. The most appropriate course of action was selected by the CEC client by considering medical safety, even for an intractable disease with intubation and an unknown patient and/or family preference. On the other hand, when the CEC team decided to “withhold,” they considered the following:

1. The veracity of the CEC client’s points of dispute

2. Accuracy in judgment of the terminal prognosis by the CEC client

3. A procedure for maintaining consensus regarding the risks and benefits of re-intubation

For the 18 cases in the “with decisional capacity” group, the consistency between the CEC team response and patient preferences were “undetermined because of ambiguous patient preference” (8 cases, 44.4%), followed by “for patient preference” (7 cases, 38.9%) and “against patient preference” (3 cases, 16.7%; Table 4).

For three of the four cases in “against patient preference” in the “partially agreed” group, the common patient preference for the three cases was to receive a medical treatment. The CEC client decided against patient preference because:

1. It can be considered an end-stage disease and end-of-life situation.

2. Despite the patient’s desire for intervention, palliative care rather than aggressive treatment was considered most appropriate.

3. Cancer treatment is harmful.

4. Patient preference is difficult to meet due to current medical regulations.

However, the CEC team decided against patient preference because:

1. The aggressive treatment would make the patient medically worse (this is also in-line with the CEC client’s opinion).

2. The reason for the patient’s insistence on aggressive treatment was unknown.

3. The risks outweighed the benefits.

4. The goals of medical care were unclear for palliative care and cancer treatment.

5. The hospital administration system does not take precedence over the patient’s best interest.

By clarifying their commitment to patient treatment and confirming that treatment would harm the patient given their condition, the CEC team was able to determine that the treatment risk was higher than its benefit to the patient, which is the goal of medical care. In addition, the CEC team pointed out that the hospital management could not serve as a contributing factor to achieving the goal of medical care.

### An example of a typical case (B) in the “with decisional capacity” group

A man in his 60s has renal cancer extending to the peritoneum. He received interferon and interleukin treatment in the urological section of another hospital but responded poorly. Upon an increase in abdominal pain, the chief physician recommended his admission to a hospice. He approved the termination of aggressive cancer treatment before hospitalization. However, after changing his mind, he expressed his wish to his family to continue cancer treatment. His family appealed for help to the CEC-client as follows: “If cancer treatment cannot be continued in this hospice because his consciousness is questionable, could you give him a placebo (e.g. saline) as a ‘drug’ instead because the continuance of cancer treatment has become the focus of his drive for life?” When a patient desires continued cancer treatment, how is the administration of a placebo seen in terms of ethics?

[CEC client’s opinion]

Interferon and interleukin treatment must be discontinued. However, it is uncertain whether administration of a placebo would be ethical.

[Advice from the CEC team]

Withdrawing cancer treatment is reasonable because the patient is terminal and it will minimize suffering.

The CEC client’s considerations regarding continued treatment were as follows:

For: patient preference is strongly in favor of continued cancer treatment; the family wishes to comply with the patient’s preference.

Against: continued cancer treatment breaches the rules of the palliative care ward; the patient is terminal and withdrawing cancer treatment is beneficial.

Therefore, administration of placebo was not considered.

The CEC team’s considerations regarding placebo use were as follows:

For: it may alleviate pain psychologically.

Against: placebo use breaches legal principles regarding informed consent; it has never been proven legally that placebo use can improve patient QOL in clinical settings; placebos cause no clinical improvement and have no life-prolonging effects on patients with terminal cancer; psychological harm could result from discovery of placebo use; and creating false hope through lies is poor medical practice.

On the basis of the patient’s condition, the CEC client judged against the patient and in favor of palliative care from a professional healthcare standpoint. Furthermore, the restrictive system in which the CEC client operates made it impossible to follow both the patient’s wishes and the requests from the family to comply with said wishes. Even though the therapeutic treatment does not benefit the patient medically, the fact that there is still treatment for a disease gives the patient a hope of life. In this instance, futility of treatment becomes an issue. In the meantime, the patient family appealed for the use of a placebo, as they wanted the patient to preserve his hope about living. Thus, another ethical issue arises as to whether healthcare professionals are justified in lying about placebo use under the guise of maintaining hope to live. In addition, healthcare professionals are obligated to provide appropriate medical care to individuals under their care, and if they are unable to provide appropriate care, then they have a duty to refer the patient to healthcare professionals who have this capacity. The CEC team approved the CEC client’s decisions to admit the patient to palliative care, conditional on the accuracy of the medical evaluation performed by the CEC client. However, the CEC team did not justify placebo use in order to adhere to the patient’s wishes of continuing aggressive treatments. Placebo use in end-of-life conditions occurs without the patient’s consent and on the assumption of deceiving the patient. If a placebo were given to a patient with decisional capacity, it would not be justified because it could violate the patient’s right to self-determination. If a placebo were given to a patient without decisional capacity, it would also be unjustified, because it would medically have no effect on the patient. Thus, the CEC team proposed a framework to guide the CEC client’s ethical judgment in order to resolve the CEC client’s confusion.

## Discussion

Although CEC clients showed some consideration of clinical ethics, this study found significant differences of opinion between CEC clients and CEC teams. First, in the cases of without/uncertain decisional capacity, the CEC team more often disagreed or withheld their opinion. This was due to different reasoning between the CEC client and CEC team. More specifically, CEC client judgments were a compromise involving the psychosocial and legal/organizational standpoints toward the case, whereas CEC team judgments were an exploration based on patient preferences and the patient’s biomedical and psychosocial best interests, which were both identified through case analysis. In deliberations, the CEC client cited not only the practical considerations of powerful parties, but also fewer logical medical considerations than did the CEC team. In contrast, the CEC team simply summarized the points of ethical consideration by the CEC team, such as the patient’s preference and best interest, in an effort to achieve an ethical clinical judgment.

The differences between the CEC client and CEC team appear to be in their differing perceptions of case-related information. In the clinical setting, patient care depends on information. The CEC client’s judgment is based on interpretation of information; they tend to acknowledge the preferences/opinions of clinically involved parties as facts. However, value judgments are based on factual information. It is problematic that the CEC clients started generating a consensus among parties’ preferences without knowing how the preferences are affected. If there are other, additional pieces of factual information, the preferences tend to change. Ethical problems develop from uncertainty among healthcare professionals as well as patients’ and their family’s perception of situational information in the clinical setting [18]. Additionally, the family’s preferences often have psychosocial influences on the patient [19], and the CEC client hope that their wish is obeyed [20-22]. Thus, to make accurate clinical judgments, professionals must review both patients’ and families’ preferences.

The legal standing of living wills in Japan is not clear. Furthermore, it may be difficult in clinical practice to judge the end stage of non-tumor-related diseases, such as interstitial pneumonia. Therefore, in healthcare settings, maintenance of physical functioning in patients with non-tumor-related disease is an indication of treatment. In other words, when a patient with end-stage non-tumor-related disease has not made a non-treatment decision clear and the family clearly desires treatment, physicians generally decide to provide treatment. In addition, medical staff members find it more psychologically stressful not to provide treatment than to provide it [23,24]. Consequently, physicians may prioritize the intention of the family who desire treatment over the patient who refuses it.

It is likely that the CEC client’s judgment is subject to influence from the patient’s and family’s preferences and expectations, and hospital/health-care-system rules. For decision making in clinical settings in Japan, physicians traditionally prefer to confer with family, not the patient [20]. Moreover, the family often desires involvement in decision making for the patient [25]. Consequently, decision making tends to more closely reflect family than patient preference [26]. In other words, healthcare providers seem to seek a balance of power between patient and family. Therefore, healthcare providers are more sensitive to the family than to the patient and tend to confirm the intentions of the family, not those of the patient. In decision making, it is necessary that the patient and the family achieve consensus regarding their intentions. However, when such consensus fails, or consensus building is not performed, intervention by the CEC as a third party may provide the best benefit for the patient.

Although clinical ethical support services are necessary, at present, such judgments have been left to the discretion of clinicians in Japan, especially since the majority of medical institutions are general and medium-sized hospitals. Such hospitals do not necessarily possess sufficient human and financial resources. A domestic survey performed by Nagao et al. in 2005 [7] showed that an ethical support system was not adequately established in hospitals, although there were many opinions that indicated that clinicians required clinical ethical support. Today, medical institutions accredited by the Japan Council for Quality Health Care are required to have some type of clinical ethics system in place; most of these systems have an ethical committee. Although there is no legal support, including legal precedent, in the establishment of the ethics committees and their judgment, ethical support services, by outsourcing, appear to be important from the perspective of meeting clinicians’ needs and establishing ethics consultations as clinical ethical support in the current healthcare setting. This was an experimental system in which clinicians’ ethical problems in healthcare settings were reviewed from a different viewpoint by scientists or researchers in medical and clinical ethics. This support can share the legal and social responsibilities mentioned above, especially the social responsibility of clinicians in healthcare settings. On the other hand, when CEC is performed in institutions, patients and their families are expected to participate in discussions regarding the best decision for the patient. In this case, final decisions are made by the patient, family, and the medical care team together, encouraging consistency of viewpoints regarding support among everyone.

CEC clients are usually concerned about their own action in legal or psychological matters because they are the ones involved in actual clinical practice. According to a study by Foglia et al. [12], ethical challenges reported by clinicians included maintaining high-quality patient care despite limited resources, as well as balancing their duty to their patients while fulfilling their obligation to stewardship of institutional resources. For patients with uncertain or no decisional capacity, the CEC client also focuses on practical factors, such as how to manage healthcare quality by providing safe patient management in the frame and course of treatment; thus, their judgments are strongly affected by medical risk management.

However, in engaging the concerned parties’ preferences, the CEC team tends to review the veracity of the CEC client’s individual medical preferences; the CEC team acknowledges that both facts and values intertwine with preferences. Therefore, the CEC team aims to make consistent judgments on critical and reflective review. According to a study by Fox [27], vignette recommendations by ethics consultants were not associated with an advance directive for all life-prolonging treatments in cases where there was no possible chance of the patient ever regaining consciousness. Their recommendations seemed to take the stance of resource allocation and/or futility. The results of our study revealed that the CEC teams were likely to “withdraw” to the CEC clients’ positions. This indicates that the CEC team addresses the following points: determining the patient’s condition or preference, determining whether the family member is a proper surrogate, and comparing interests with risks to estimate the veracity of patient preference. The CEC team uses the family as a source of information to estimate the intentions of surrogate and patient preferences. Consequently, the CEC team expects that eliciting patient preferences from patients as much as possible is a central challenge. The previous points should be considered important, because ethics consultation is a style of outsourcing.

The CEC team examined how to ethically integrate, influence, and understand clinical information; for example, they identified factors that influenced the patient’s concern about the treatment, and whether or not the patient fulfilled his/her preference by accepting treatment. It seems that the CEC team assesses the true goal and purposes of the patient’s appeal. The CEC team also interprets legal and ethical standards to examine whether the healthcare provider’s actions are consistent with unbiased consideration, such as distributive rights based on reasons and procedural rights by healthcare providers. The CEC team’s goal is to provide the maximum possible benefit to patients; therefore, it devised procedures to achieve agreement and advice within the frame of the points discussed among members. This is the process by which ethical judgments are made. Perspectives described based on disagreed-upon and withheld information generate ethical discussions between consultants and clients. In other words, clients allow CEC consultants to exercise their expertise in ethical problem solving.

The results of this study revealed a striking pattern regarding treatment recommendations for patients with decisional capacity; both the CEC client’s and the CEC team’s judgments went against clear patient preferences. A major focus within the medical ethics field has been to respect patient autonomy. However, CEC’s advice ran contrary to patient preference, which is remarkable in the field of clinical ethics.

A common source of conflict in these cases is that aggressive treatment for cancer was expected to be harmful to the patient. Such conflicts occurred between patients and healthcare providers during the start of the shift to end-of-life palliative care in each case within the “against patient preference” in the “partially agreed” group. The CEC client’s medical preference was against aggressive treatment and its medical limitations; in the CEC client’s opinion, the patient’s best interests would be served by a shift from aggressive treatment to end-of-life palliative care. In the patient’s mind, being treated was a means of living, and treatment withdrawal meant dying. This type of situation arises because current medical care in Japan has been specialized and separated by stage of disease, such as the division between hospital and hospice. According to studies from the US [28,29], medical professional integrity and judgment were influenced by factors such as resource allocation and/or hospital size. In a similar manner, Japanese clinicians made decisions as professionals, committing to the management of high-quality healthcare despite limited resources. Although medical care should be a part of the patients’ lives and although some concomitant suffering is normal, the CEC client is more likely to view the patients’ healthcare and experience according to the biomedical model.

However, the CEC team considers both aggressive treatment and palliative care to be continuous patient care [30,31]. The appropriateness of a certain invasive treatment is judged according to whether the benefits exceed the risks. The CEC team likely views the patient’s suffering from an illness-related rather than disease-related perspective. The CEC team’s goal is to determine the type of healthcare that will best serve the patient’s interests. The CEC team could fulfill a need by examining the veracity of information and recognizing values with facts. Thus, the CEC team judgments were critical and reflective, while the CEC client judgments were situational and rather practical.

### Limitations

In this study, there were a number of different perspectives and reasons for professionals’ judgments. Nevertheless, CEC clients gave relatively little information on cases. The following are the possible reasons as to why CEC clients do not share all of their information: (1) while they have the information, they do not realize that it is necessary for making decisions; or (2) such information is not collected at the sites. Ethics consultants gave advice with such points in mind. Moreover, the CEC team needed to make a judgment with a limited amount of information; that is why many cases in this study were in the withholding group.

The CEC team dealt with information from CEC clients who were anonymous. They were unable to obtain further information regarding the CEC client’s judgment and/or their request for the CEC. In addition, the CEC team made their recommendations according to limited clinical information. For these reasons, we did not ask either of these parties for additional information. As a result, details regarding the factors that influenced the CEC team’s conclusions are unclear. This study aimed to analyze the content of the process in the context of the document, not within the context of priority or significance. We hope to improve on these methods so that the process of consensus development may be examined in the future.

This study has the following additional limitations. First, CEC clients may not be representative of healthcare providers in clinical practice because they were highly interested in clinical ethics. Because the CEC team’s requested documents were used for this study’s analysis, not all of the CEC clients’ thoughts and judgments were included. The second limitation refers to the ethics consultation process; the basis of the advice from CEC teams may vary depending on the members. We could not clarify whether the content requested was associated with the client’s background or the CEC team members’ backgrounds and advice. The diversity of the team in the consensus-building process could also not be analyzed because the content of the email meeting could not be collected as data. However, the bias inherent in the advice of the ethics consultants is thought to be minimal, because CEC is provided not by a single individual, but by a team of various specialists from different fields of expertise. Cases will continue to be handled by CEC in the future. These should be accompanied by reports that will generate additional criteria for advice, judgment, particularity, and universality of care. We also expect to be able to study differences among the CEC members’ ethical backgrounds by their profession. In addition, if ethics judgments are universal, they should not be affected by country or culture. Therefore, it will be necessary to conduct an international comparative study on ethics consultants’ means of understanding the patient, as well as their advice to the patient and their practical ethical judgments.

## Conclusions

In this study, we examined the differences in ethical judgments between the CEC client and CEC team. Differences in opinions were found between cases with uncertain and without decisional capacity. The reasons leading to the judgments differed between the CEC clients and the CEC team. The CEC team’s judgments were focused on critical and reflective bases for veracity; the CEC clients’ were based on situational and practical factors. Further analysis regarding the rationale of ethics consultants and comparison of healthcare professionals regarding this matter will improve the wellbeing of patients and cultivate the professional expertise of ethics consultants.

### Ethics approval

This project was approved by the Ethical Review Board of the Kumamoto University, School of Medicine until 2011. The study was approved by the ethics committee of Kobe University, Graduate School of Health Sciences.

## Competing interests

The authors declare that they have no competing interests.

## Authors’ contributions

All authors (NN, YK, and AA) designed and conducted this study, and collected the data. NN and YK analyzed the data with supervision from AA. All authors participated in writing the manuscript and approved the final version.

## Pre-publication history

The pre-publication history for this paper can be accessed here:

http://www.biomedcentral.com/1472-6939/15/19/prepub
